# Values and National Identification in Minority and Majority Youth: Longitudinal Multi-Study Findings

**DOI:** 10.1007/s10964-024-01965-0

**Published:** 2024-03-14

**Authors:** Maya Benish-Weisman, Ella Daniel, Einat Elizarov, Noga Sverdlik, Peter F. Titzmann

**Affiliations:** 1 The Hebrew University of Jerusalem, Jerusalem, Israel; 2 Tel-Aviv University, Tel Aviv-Yafo, Israel; 3 The University of Haifa, Haifa, Israel; 4 Ben Gurion University, Beersheba, Israel; 5 Leibniz University Hanover, Hanover, Germany

**Keywords:** Values, Identity, National identification, Adolescents, Minority, Majority

## Abstract

Collective identification is vital for adolescents, fostering well-being and connection, but scant attention has been given to drivers of national identification and their contextual variations in youth. To address this, two longitudinal studies examined how values, as guiding goals defining what individuals consider important in their lives, relate to the trajectory of national identification in majority and minority youth. Study 1 (*N* = 568; M_age_ = 16.24, SD = 0.71) and Study 2 (*N* = 678; M_age_ = 13.78, SD = 0.73) focused on majority youth (Jewish-Israelis), while Study 2 also included minority (Arab citizens of Israel). The findings highlight values as important motivators of national identification over time. Conservation values, emphasizing the preservation of the status quo and a preference for stability, were prominent motivators for the majority of adolescents. In contrast, power values, which center around climbing the social ladder and accumulating wealth, held greater significance among their minority counterparts; however, both sets of values correlated with increased national identification. The discussion touches on motivations underlying national identification, their contextual diversity, and implications for future studies.

## Introduction

Cultural or ethnic identification has an important role in adolescents’ social development and mental health. Being part of a larger group helps individuals feel connected and gives them a sense of belonging and support (Smith & Silva, 2011). However, very little is known about the motivators of group identification, especially among adolescents. What factors motivate adolescents’ identification with a cultural group? Adolescents’ values may play a central role in this type of identification. The personal values framework (Schwartz, 1992) defines values as basic abstract motivations that vary in importance and constitute a central aspect of the self. They relate to the ways people perceive the world around us and affiliate with different groups (Roccas et al., 2010). Adolescence is a time of identity and social change; thus, values and social belonging and their relations might play a special part in this period. Nevertheless, the few studies that have focused on these relations have considered only adults, ignoring the developmental aspect. Furthermore, scant attention has been paid to how context might affect the relations between values and national identification. Adolescent values may be particularly apt predictors of national identification but this may differ across ethnic groups. Two independent longitudinal studies were conducted to examine the role of values in predicting change in national identification among majority youth, Jewish-Israelis (Studies 1 and 2), and minority youth, Arab citizens of Israel (Study 2).

### Values and National Identification during Adolescence

Adolescence is a particularly interesting time to study identity development, because it is a major developmental task at this stage in life (Havighurst, 1972; Kroger, 2006) and because it is a rather complex endeavor with changes in nearly all spheres of life including the formation of two types of identities: the personal and the collective (Eccles, 2009; Schwartz et al., 2008). The first type, *personal* identity refers to developing a sense of uniqueness – identifying what makes a person different and special, and answering questions such as ‘Who am I?’ and ‘What is important in my life?’ During adolescence, there is a notable advancement in brain development, leading to improved decision-making abilities (Crone & Van Der Molen, 2007) and the development of meta-cognition, which involves the capacity to assess one’s own thoughts (Kuhn, 2009; Kuhn, Cheney, & Weinstock, 2000). As adolescents gain the ability to think abstractly beyond specific situations (Dumontheil & Blakemore, 2012), they refine and improve their guiding goals and values. The personal values system can be seen as a manifestation of a unique identity, one that highlights what motivates individuals and what they aspire to achieve.

The second type – *collective* identity – entails individuals’ sense of belonging to social groups, defined by their social roles (e.g., student, athlete) and demographic characteristics (e.g., gender, nationality). This identification answers the question: ‘Who am I as a member of my group and in relation to other groups?’ Groups have a significant importance during adolescence, as brain development makes adolescents extensively sensitive to the social environment (Laursen & Veenstra, 2021; Telzer et al., 2022). Moreover, as adolescents gain increased in-dependence from their parents (Branje, 2018), groups offer a set of norms which provide a benchmark for what they can expect from others and how to behave within a social context. Therefore, identification with groups is associated with perceptions, attitudes, and behaviors that are in accordance with group norms and manifest themselves in individuals’ collective identity (Hogg & Abrams, 2007; Tajfel, 1974). Based on the importance of these factors at this age, it is surprising that no study addressing how values relate to collective identity has been conducted among adolescents. The studies described herein aimed to fill this gap.

Values preferences as part of personal identification and identification with the nation as collective identification, (Eccless, 2009) have rarely been studied together, although there is a theoretical link. Identification with groups satisfies basic human needs and motivations (Correll & Park, 2005; Roccas et al., 2010). It can fulfill the need to avoid uncertainty, the motivation to avoid death-related anxiety (Greenberg et al., 1990; Solomon et al., 1991), the desire to belong and feel embedded in society (LaFromboise et al., 1993), and the motivation to enhance one’s self-image (Tajfel & Turner, 1979, 1986). In addition, because different groups potentially satisfy different motivational needs, by identifying with various groups, adolescents can express the relative importance of different motivations (i.e., preferences for certain values). For example, identification as an athletic team member may satisfy a personal value of excellence, while identification as a member of a friend’s group may satisfy a personal value of care. Therefore, group identification is also related to the formation of a personal identity. This implies that an individual’s identification with a group will be stronger the more the group fulfills that individual’s motivational needs and the more the individual gives importance to this same motivation.

Arguably, values preferences may contribute to changes in identification as a national group member (i.e., national identification), and these relations may differ by contextual factors. More specifically, values preferences may predict differences in identification with the nation (aspect of collective identity) across time for minority and majority youth.

### Values as a Motivational Factor Explaining Identification with National Group

**Fig. 1:**
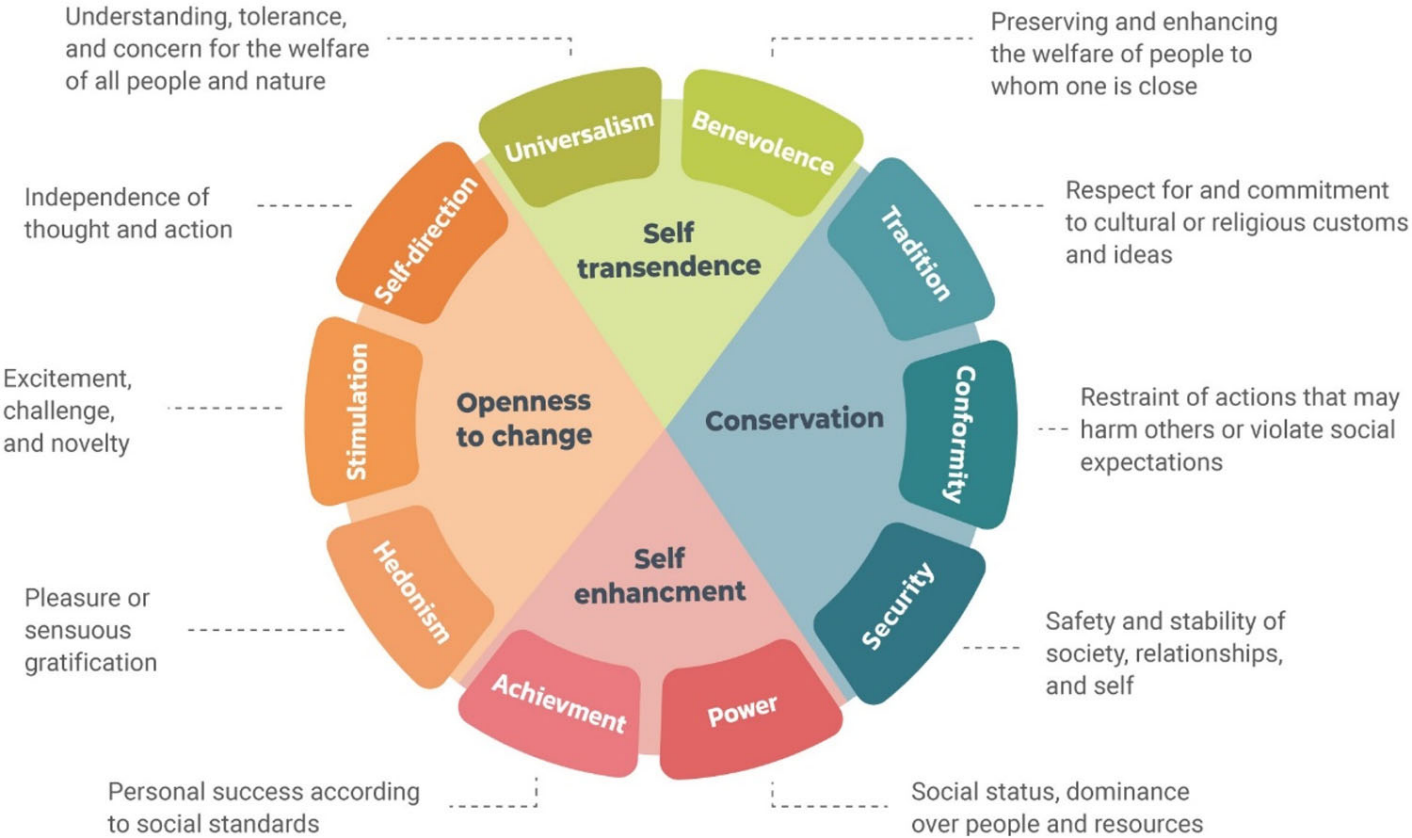
The Schwartz Basic Values model

Personal values are guiding goals defining what individuals consider important in their lives. Values serve as criteria for evaluating the self and others, and they impact attitudes, behaviors, and perceptions (Schwartz, 2012). Although all our values are important, different adolescents will endorse different values. For example, some will endorse social justice and equality, while others pursue enjoyment. Personal values tend to transcend specific actions or situations. That is, an adolescent who attributes importance to openness to change will tend to befriend new peers at school and may enjoy extreme afterschool activities such as paragliding. Schwartz’s Basic Values Theory (Schwartz, 1992) identifies ten values, organized in a circular structure, wherein adjacent values share similar underlying motivations, and opposing values may be contradictory. These ten basic values can be arranged on two bipolar dimensions (see Fig. 1). One dimension contrasts values of conservation with values of openness-to-change. Conservation focus on the preservation of the status quo, preference for stability, and adherence to social and cultural expectations and rules, including values of tradition, conformity, and security. Values of openness-to-change focus on embracing change and autonomy, including values of self-direction, hedonism, and stimulation. The second dimension contrasts values of self-transcendence with values of self-enhancement. Self-transcendence values focus on the welfare of others, and they include values of universalism and benevolence. Values of self-enhancement focus on the promotion of self-interests and are comprised of values of power and achievement.

The universality in content and structure of the motivational continuum in Schwartz’s values framework has been validated in over 70 cultures (Sagiv & Schwartz, 2022; Schwartz, 2011), making this framework particularly useful to compare broad motivations across diverse cultural groups (Roccas et al., 2010). Moreover, because the values theory describes a full spectrum of motivations, it allows researchers to examine a variety of motivations and identify potential differences between groups, in this case, differences in motivations underpinning national identification. Finally, the values theory was not developed within a group processes framework; therefore, relations between values and national identifications are not likely to be explained by shared content or measurement (Roccas et al., 2010).

### Motivation to Identify with a National Group across Contexts

Values can be a powerful motivational force to help people achieve their goals and fulfill them through identification with a specific social group (Roccas et al., 2010). However, it is not known how values serve as motivational forces in identification with the nation, whether these relations exist across time, and if it should be expected them to be similar for majority and minority groups.

In most cultural settings, citizens are expected to identify with the nation as an expression of patriotism. Accordingly, identification with a nation is often seen as a positive, normative characteristic (Bar-Tal, 1993). For people who are part of the majority group, this type of normative identification can provide a sense of safety and stability, as well as a sense that they are living up to widely accepted social standards. Thus, identification with the nation enables the expression of *conservation values* that focus on stability, adherence to expectations, and maintenance of the status quo. In contrast, those who are *open to change*, and value independence and freedom may be more reluctant to identify with groups in general, and with broad groups such as the nation in particular, because such groups may threaten their sense of autonomy and freedom. In support of this theory, a previous study focusing on a majority group found conservation values were positively related to national identification, and openness-to-change values were negatively related (Roccas et al., 2010). Additional support for this pattern of associations can be found in studies exploring the links between values and political ideologies. Specifically, a growing body of research shows that conservation values are positively and openness-to-change values are negatively related to right-wing political attitudes and strong expressions of patriotism (e.g., Piurko et al., 2011; Schwartz et al., 2010; see a review in Sagiv & Schwartz, 2022). These studies also point to universalism values as an additional value to consider in the present context. Specifically, universalism values that focus on promoting the welfare of all humans, ingroups and outgroups alike, are negatively associated with right-wing attitudes, such as blind patriotism, and positively associated with the acceptance of outgroups and the endorsement of equality for all. Thus, a more universalistic view would suggest a preference for fewer national-related boundaries, and it should be expected a negative association between universalism and national identification.

This study went beyond simply testing relations between values and national identification. Values may not only relate to national identification concurrently but may also explain *changes* in national identification across time. These dynamic associations can be especially expected to be found in adolescents, who are at a life stage when values and group identification may be prone to change as part of their growing up. In particular, need fulfillment and social group belonging drive developmental changes so that conservation values likely predict an increase in national identification and openness-to-change and universalism values may predict a decrease in national identification over time among adolescents.

Given the growing diversity in today’s societies (Titzmann & Jugert, 2019), it is important to develop an understanding of the motivational profiles of national identification of different social groups, because all developmental processes in diverse societies must consider adolescents’ race or ethnicity (Yip et al., 2019). Minority adolescents are confronted with different cultural norms and rules across contexts, such as the family and the school, and negotiate a sense of belonging to either or both heritage and host cultural groups (Jugert et al., 2018). Roccas and her colleagues (2010) suggested that conservation values are motivators for national identification in the dominant group, as national identification supports stability and reduces uncertainty. But they acknowledged that national identification can provide a sense of uniqueness among immigrants, marking them as different from their ingroup, other immigrants. Furthermore, when the individual is affiliated with a minority group that is marginalized by the majority, identification with the nation is less likely to be motivated by the search for stability, as stability will entail maintenance of the marginalization within the national group.

The degree of identification with nation is known to vary by context, but there is little knowledge about motivations underlying the national identification of majorities and minorities. National identification among minorities may be related to a desire to advance their personal interests within society. For an individual from a marginalized minority group, belonging to the majority group may promote that individual’s status; therefore, the desire to gain power and social status may enhance minorities’ identification with the majority group. This is supported by previous research finding self-enhancement values (such as power) predicted identification with high-status groups (Roccas, 2003). Universalism values focus on equality, justice, and inclusiveness, and as such, they stand in contrast to self-enhancement values. Individuals who endorse these values have a low need for status and self-promotion. Furthermore, they are more likely to be sensitive to the violation of justice and equality represented in the marginalizing attitudes expressed by the dominant group and the nation. Thus, universalism values may be negatively related to national identification among minorities.

The motivation for identification with a nation should not be examined only from a general minority versus majority perspective; the specific context has to be considered. A meta-anlysis found identification with both majority and minority cultures is important for adjustment and well-being (Nguyen & Benet-Martínez, 2013). However, some contexts might challenge these findings. The two studies described here focused on Jewish-Israelis (majority) and Arab citizens of Israel (minority). *Arab citizens of Israel* are Palestinians whose families lived in what is now the State of Israel before its foundation. They comprise 21.1% of the Israeli population; a majority practice Islam (Horenczyk & Ben-Shalom, 2006; Israel Central Bureau of Statistics, 2022). Although there are growing collaborations between the Jewish and Arab communities in Israel (e.g., Ben-Lulu & Feldman, 2022), as a result of the ongoing conflict, including terror attacks, the Arab citizens of Israel are perceived by some members of the majority group as a danger (Litvak-Hirsch, Bar-On, & Chaitin, 2003) and their loyalty is questioned (Hammack, 2010). Through the years of the conflict, there has been a growing distrust between the communities. Arab citizens of Israel are subject to suspicion and violence at the hands of the police and army, and they experience discrimination on personal and institutional levels. On average, they also experience lower income, higher unemployment rates, and a greater proportion of residents reliant on social security payments compared to their Jewish counterparts. (Feasel, et al., 2021). As Israel was founded as a Jewish state, the prominence and prevalence of Jewish culture may lead to non-Jewish individuals feeling that there is not enough space for their own cultural expression. This is likely to impact their feeling of belonging, and they may even be antagonistic to the state of Israel (Hammack, 2010). Arab adolescents are likely to be affected by these factors, and this, in turn, will affect their national identification. National identification may not relate to a desire for security, but it may serve as a tool for social climbing and entering the dominant Jewish society. Similar processes were found among Muslim Americans following 9/11 (Fine & Sirin, 2007).

## The Current Study

Personal values priorities may predict change in national identification among adolescents. Moreover, being a majority or minority group member may play an important role in the process of national identification formation. Specifically, different motivational mechanisms may predict identification change and formation among majority and minority groups in Israel. However, these hypotheses have not been tested until today. This study tests four hypotheses. First, conservationvalues will predict an increase in national identification among majority adolescents. Second, openness-to-change values will predict a decrease in national identification among regardless of group affiliation. Third, universalism values will predict a decrease in national identification among adolescents regardless of group affiliation. Fourth, power values will predict an increase in national identification among minority adolescents.

The hypotheses were tested in two longitudinal studies. Study 1 was conducted among Jewish adolescents in Israel in grades 10 and 11 at the first time point; it tested hypotheses 1–3 in a majority group. Study 2 was conducted among Jewish and Arab adolescents in Israel in grade 8 at the first time point; the study tested all four hypotheses.

## Study 1

The aim of Study 1 was to investigate longitudinal associations between values and national identification among majority adolescents. This study adds to a growing body of research on national identification among majority groups and fills a gap in knowledge about the association between values and national identification across time. Conservation values were hypothesized to predict an increase in national identification and openness-to-change values and universalism were hypothesized to predict a decrease in national identification among majority adolescents.

### Method

#### Participants and procedure

The study included *N* = 568 (T1) adolescents from Jewish public schools in Israel. Tenth and 11^th^-grade students were approached through their school at T1 (*M*_age_ = 16.24, *SD* = 0.71, females = 55.5%). Participants were approached again the following year. The schools were from different urban areas of Israel. All participants and their parents were native Israelis. Participants reported their mothers’ and fathers’ highest level of education: elementary, 3%, 5.4%; high school, 63.3%, 61.5%; university, 26.9%, 25.7%, for mothers and fathers, respectively. Values were missing for 7.4% of mothers and 6.5% of fathers.

Schools were randomly sampled from a list of schools in two major urban centers and in towns populated by a large percentage of immigrants according to the Israel Central Bureau of Statistics. Schools were approached by telephone, and six agreed to participate. Parents received consent forms before school sessions. In each school, questionnaires were distributed by trained research assistants. They explained the instructions for the questionnaires and answered questions. Questionnaires were translated into Hebrew, using back-translation procedures. The questionnaires were anonymous, and participation was voluntary. The local ethical review board in the Tel- Aviv University approved the study.

As part of the requirements of the Ministry of Education, to protect the privacy of the participants, the list of participants was left at the schools, and only some could be retrieved. Therefore, it was impossible to match all the T2 participants to T1 participants. As a result, the attrition rate was 65.6%. That is, 198 adolescents participated at both time points, and 370 adolescents participated only at T1. T-tests of participants who took part in both waves and participants who took part only in the first wave revealed no differences between the groups in gender, national identification, or in conservation, openness-to-change, universalism, and power values. One significant difference between groups was that participants who completed both assessments were older (*M* = 16.33, *SD* = 0.67) than participants who dropped out before completion (*M* = 16.19, *SD* = 0.72), *t*(565) = 2.36, *p* = 0.02).

### Measures

#### Personal values

Adolescents’ values were assessed using a short version (25 items) of the Portrait Values Questionnaire (PVQ; Schwartz et al., 1999, 2001). Each item in the questionnaire describes a person’s goal, aspiration, or wish, pointing implicitly at the importance of a single broad value. For example, the item ‘He thinks it is important that every person in the world should be treated equally. He believes everyone should have equal opportunities in life’ describes a person for whom universalism values are important.

Participants indicated ‘how much each person is or is not like you’ for each item. They checked one of six boxes ranging from ‘not at all like me,’ to ‘very much like me.’ Thus, participants’ own values were inferred from their self-reported similarity to people described in terms of particular values. The similarity judgments were transformed into a six-point numerical scale. Indices for each value were computed by averaging all items measuring the value. In order to control for scale use, the scores were centered around each participant’s mean, in a procedure recommended by Schwarz (1992). The PVQ has been shown to be suitable for use with children and adolescents (Bubeck & Bilsky, 2004; Knafo & Schwartz, 2003; Schwartz et al., 2001). The scale was originally written in Hebrew and was measured and validated among Israeli adolescents (Schwartz et al., 2001), and also among Arabic-speaking adolescents (Schiefer et al., 2010).

Cronbach’s alpha was 0.76 for conservation values (seven items), 0.74 for openness-to-change values (seven items), 0.55 for universalism values (three items), and 0.58 for power values (two items) at T1 and 0.76 for conservation values, 0.76 for openness-to-change, 0.57 for universalism values, and 0.59 for power values at T2, for the same number of items as T1. As this measure covers a large range of meanings of each value with few items, for some values (power and universalism), the alpha’s values were relatively low. Nevertheless, these reliabilities were within the range of previous values studies (Schwartz, 2005), in which other methods were employed to establish the validity of the measures. Furthermore, adolescent questionnaires need to be as short as possible to keep the participants motivated. Accordingly, some scales included a small number of items, naturally resulting in low reliabilities (see Spearman–Brown prediction formula).

#### National identification

Participants’ national identification was measured by three items on the centrality-to-identity scale from the Identification Questionnaire created by Roccas et al., (2008). Participants were asked to rate their agreement with three statements on a 6-point Likert-type scale, from 1 ‘strongly disagree’ to 6 ‘strongly agree’. A sample item was: ‘Being Israeli is an important part of my identity’. Cronbach’s alpha was 0.94 for T1 and 0.94 for T2. The identification questionnaire was developed and validated in Hebrew (Roccas et al., 2008) and Arabic (Daniel et al., 2016).

#### Demographics

Gender was measured. Socio-demographic status was calculated based on the average score of reported parents’ higher education (elementary, high school, and university).

### Results

#### Associations between values and national identification

Table 1 presents the mean and SD scores and the concurrent associations of the study variables. The results show that in line with our hypothesis, the conservation values of Jewish adolescents at T1 were related to national identification at T1 and T2. No significant associations were found between conservation values at T2 and national identification measured at T1 and T2. In addition, universalism values at T1 were negatively related to national identification at T2, and power values at T1 were negatively associated with national identification at both times. Openness-to-change values were not associated with national identification at either time point.

**Table 1:** Descriptive statistics and concurrent relations between Study 1 variables among Jewish adolescents

	Mean	SD	Gender	SES	Age	Conservation		Openness		Universalism		Power		National ID	
						T1	T2	T1	T2	T1	T2	T1	T2	T1	T2
SES			−0.02												
Age	16.24	0.71	0.06	−0.10*											
Conservation															
T1	3.75	0.59	−0.05	0.06	0.05										
T2	3.81	0.60	−0.06	0.03	0.13	0.63***									
Openness															
T1	4.27	0.56	0.05	−0.07	−0.07	−0.68***	−0.49***								
T2	4.22	0.60	0.06	−0.00	−0.10	−0.43***	−0.71***	0.60***							
Universalism															
T1	3.87	0.71	0.11**	0.02	−0.02	−0.04	0.07	−0.25***	−0.17*						
T2	3.92	0.70	−0.03	0.02	−0.13	−0.00	0.03	−0.17*	−0.25***	0.46***					
Power															
T1	3.16	1.10	−0.19***	−0.04	0.01	−0.27***	−0.31***	0.06	0.07	−0.31***	−0.18*				
T2	3.10	1.03	-0.10	0.02	−0.12	−0.16*	−0.33***	0.07	0.03	−0.24***	−0.35***	0.56***			
National ID															
T1	5.44	0.95	0.05	0.04	0.04	0.10*	0.05	−0.03	0.03	0.04	0.09	−0.15***	−0.10		
T2	5.57	0.87	−0.08	−0.31***	0.06	0.15*	0.12	0.11	−0.05	−0.14*	−0.08	−0.20**	−0.13	0.37***	

*National ID* National Identification, *T1* Time 1, *T2* Time 2

^*^p < 0.05; ^**^p < 0.01; ^***^p < 0.001

#### Associations between values and national identification across time among Jewish majority adolescents

The analyses were conducted using Mplus 8.1 (Muthén & Muthén, 2017). Missing data ranged from 0.2% to 65.6%. Little’s missing completely at random (MCAR) test was not significant, *χ*^*2*^ (33) = 40.43, *p* = 0.18, indicating the data might be MCAR. Therefore, our estimation method was maximum likelihood with robustness to nonnormality (MLR).

To examine the longitudinal relationships between values and national identification, cross-lagged panel path analyses were performed for each value separately (Kenny, 1975). This analysis enabled the examination of the association between values and future national identification with control of national identification at T1. To the best of the authors’ knowledge, the study was the first to examine these relations longitudinally among adolescents; therefore, the analysis permitted the exploration of the directivity between national identification and future values. Three fit indices were used to determine the adequacy of the model fit: the comparative fit index (CFI; Bentler, 1990), root mean square error of approximation (RMSEA; Kline, 2011), and the standardized root-mean-square residuals (SRMR; Hu & Bentler, 1999). Kline (2011) has suggested that excellent model fit is achieved by CFI ≥ 0.95, RMSEA ≤ 0.06, and SRMR ≤ 0.06, with models resulting in CFI > 0.90, RMSEA < 0.08, and SRMR < 0.09 considered adequate fit. Because *χ*^*2*^ is influenced by the sample size, it was not utilized to determine the models’ fit (Marsh et al., 1988). Our final models reached excellent fit (CFI = 1, RMSEA = 0.00, SRMR = 0.02 for conservation values; CFI = 1, RMSEA = 0.01, SRMR = 0.03 for openness-to-change values; *CFI* = 0.99, RMSEA = 0.03, SRMR = 0.01 for universalism values; CFI = 0.99, RMSEA = 0.03, SRMR = 0.01 for power values).

Table 2 presents the results for the path models of the associations between values and national identification across time after controlling for gender and reported SES. Our examination of the directionality of the paths between the specific values and national identification revealed that in line with the first hypothesis, conservation values were positively related to future national identification. That is, higher levels of conservation values related to an increase in national identification across time. Contrary to the second, no associations across time were found between openness-to-change values and national identification. However, as predicted by the third hypothesis, universalism values were negatively related to future national identification; that is, higher levels of universalism values were related to a decrease in national identification across time. An additional finding was that power values were negatively related to future national identification; that is, higher levels of power values were related to a decrease in national identification across time. A concurrent marginal positive association was also observed between conservation values and national identification at T1 (*p* = 0.051). No concurrent associations appeared between openness-to-change or universalism values and national identification. Finally, power values were negatively related to national identification at T1. The analysis was rerun without the controls, with similar findings.

**Table 2:** Model results linking values to national identification across times 1 and 2 among Jewish adolescents: Study 1

	Conservation		Openness		Univeralism		Power	
	β	SE	β	SE	β	SE	β	SE
Auto-regressive paths								
Values T1-> Values T2	0.62***	0.05	0.61***	0.05	0.49***	0.08	0.60***	0.05
National Identification T1-> National Identification T2	0.39***	0.10	0.38***	0.11	0.41***	0.10	0.37**	0.11
Cross-lagged relations								
Values T1 -> National Identification T2	0.14*	0.06	0.08	0.06	−0.18*	0.09	−0.15*	0.08
National Identification T1 -> Values T2	0.03	0.09	−0.01	0.10	0.05	0.08	0.01	0.06
Concurrent relations								
Values T1 <-> National Identification T1	0.11	0.05	−0.03	0.05	0.03	0.06	−0.15**	0.04
Values T2 <-> National Identification T2	0.02	0.12	−0.14	0.12	−0.03	0.07	−0.05	0.10

*Openness* openness-to-change values, *T1* Time 1, *T2* Time 2

^*^p < 0.05; ^**^p < 0.01; ^***^p < 0.001

Thus, the results supported the first and third hypotheses. Specifically, conservation values positively and universalism negatively predicted future national identification. However, the second hypothesis was not supported. No associations were found between openness-to-change values and national identification among Jewish adolescents in Study 1. Study 2 aimed to replicate these findings in another Jewish majority group and test the third and fourth hypotheses in the context of a marginalized minority group, Arab citizens of Israel.

## Study 2

Results of Study 1 showed that conservation and universalism values predicted national identification among majority youth. However, as the sample was restricted to majority youth, Study 1 could not test whether the association between values and national identification varies as a function of context (whether the individual belongs to the majority or minority). Furthermore, to measure values, the study used a short version of the values questionnaire. Therefore, some values (e.g., power and universalism values) included a small number of items to measure each construct. This might be the reason for the relatively low reliability of these measures. To extend the research, Study 2 added a minority group and expanded the values measure to the full version. Values and national identification may change more slowly, as they are deep cultural aspects (Hall, 1976), relatively resistant to change. Therefore, Study 2 expanded the time gap between the two assessments to two years to examine longer-term effects. The associations between conservation and universalism values and national identification in the majority group were predicted to be replicated), and the second hypothesis contention that openness-to-change values would be negatively associated with future national identification among majority youth was retested. In addition, the hypotheses, whereby universalism values would be negatively and power values positively associated with national identification across time, were tested in a minority youth sample.

### Method

#### Participants and procedure

The study included *N* = 678 adolescents (51.2% girls) from public schools in the north of Israel. They belonged to two main cultures: Jewish (41.5%) and Arab Israeli born. Among the Arab students, 98% identified as Muslim. The students were approached through their schools; at T1, they were in the 8^th^ grade (*M*_age_ = 13.78, *SD* = 0.73), and at T2, they were in the 10^th^ grade. The participants in both groups and their parents were born in Israel. Participants reported their mothers’ and fathers’ highest level of education: elementary, 3.7%, 5.3%; high school, 37.1%, 41.9%; university, 34.2%, 27.6%, for mothers and fathers respectively. Values were missing for 25% of the mothers and 25.1% of the fathers.

Eight schools in the north of Israel were approached by telephone; five agreed to participate. Consent forms were sent to the parents of all adolescents; only those adolescents whose parents gave consent for their children to participate (over 95%) completed the questionnaires. Questionnaires were translated into Hebrew and Arabic using back-translation procedures. They were distributed by trained research assistants during a class session that lasted approximately 45 min. The research assistants explained the questionnaires’ instructions and answered questions while the students answered the questionnaires. After two years, the participants were approached again and completed the same questionnaires. For their participation, students received small, attractive incentives (novelty pens or pencils). The local ethical review board in University of Haifa approved the study.

Attrition rate was 36%. T-tests comparing participants who took part in both waves and participants who took part only in the first wave revealed significant differences in the study variables and demographics: participants who completed both assessments reported lower Israeli identification (*M* = 3.78, *SD* = 1.77) than participants who dropped out before completion (*M* = 4.28, *SD* = 1.63), *t*(621) = −3.58, *p* < 0.001). They also reported higher importance of conservation values (*M* = 3.86, *SD* = 0.44 vs. *M* = 3.75, *SD* = 0.49, *t*(671) = 3.03, *p* = 0.003) and lower importance of power values (*M* = 2.66, *SD* = 1.14 vs. *M* = 2.94, *SD* = 1.09, *t*(671) = −3.22, *p* < 0.001). Furthermore, the group of participants who completed both assessments had a significantly higher proportion of girls than boys (*Χ*^*2*^(1) = 41.05, *p* < 0.001) and a higher proportion of Arab adolescents than Jewish adolescents (*Χ*^*2*^(1) = 8.32, *p* = 0.004) compared to the group of participants who dropped out before completion.

### Measures

#### Values

Adolescents’ values were assessed using the full version of the measure described in Study 1 (Portrait Values Questionnaire; PVQ; Schwartz et al., 2001). This measure includes 40 items. Cronbach’s alpha was 0.85 for conservation values (13 items), 0.81 for openness-to-change values (ten items), 0.75 for universalism values (six items), and 0.60 for power values (three items) at T1 and 0.84 for conservation values, 0.84 for openness-to-change values, 0.77 for universalism values, and 0.60 for power values at T2 (note: the study used the same number of items at both times). As in Study 1, the scores were centered around each participant’s mean, in a procedure recommended by Schwarz (1992) to control for scale use. The questionnaire was measured and validated among Hebrew-speaking and Arabic-speaking Israeli adolescents (Knafo et al., 2008).

#### National identification

Participants’ national identification was measured using the Inventory of Black Identity (MIBI; Sellers et al., 1997), adapted to Israeli identity (e.g., centrality: ‘Being an Israeli is an important reflection of who I am’; private regard: ‘I am happy that I am Israeli’). Items were rated on a 7-point Likert scale, from 1 ‘strongly disagree’ to 7 ‘strongly agree’. This measurement replaced the national identification three-item scale used in Study 1 (Roccas et al., 2008). The MIBI was developed to assess the racial identity of African-Americans, but has been validated among other ethnic groups (e.g., Kiang et al., 2006), including Hebrew-speaking Israelis (Benish-Weisman, 2016; Sher-Censor et al., 2018). Ten positively phrased items from the centrality and private regards subscales were included in the analysis, as an initial factor analysis revealed the negatively phrased items loaded on a separate factor. Cronbach’s alpha was 0.94 at T1 and 0.92 at T2.

#### Demographics

Gender was measured. Socio-demographic status was calculated based on the average score of reported parents’ higher education (elementary, high school, and university).

### Results

#### Associations between values and national identification

Table 3 presents the mean and SD scores and the concurrent associations of the study variables. Among Jewish adolescents, as in Study 1, and in line with the first hypothesis, conservation values were positively related to national identification at both times. In addition, as in Study 1, and in line with the third hypothesis, universalism values were negatively related to national identification concurrently at both time points for majority youth (but not for universalism values at T1 and national identification in T2). In line with the second hypothesis (but inconsistent with Study 1), openness-to-change values were related negatively to national identification at T1. Finally, power values were negatively related to national identification at T1. Among Arab adolescents, substantiating the second hypothesis, at T2 openness-to-change values were negatively related to national identification. In line with the fourth hypothesis, power values at T1 and T2 were positively related to national identification at T2, and power values at T1 were positively related to national identification at T1.

**Table 3:** Descriptive statistics and concurrent relations between values and national identification among Jewish and Arab adolescents

		Mean	SD	Gender	SES	Conservation		Openness		Universalism		Power		National ID	
						T1	T2	T1	T2	T1	T2	T1	T2	T1	T2
SES	Jewish			0.03											
	Arabs			−0.08											
Conservation															
T1	Jewish	3.69	0.49	−0.12*	−0.06										
	Arabs	3.94	0.42	0.06	−0.08										
T2	Jewish	3.70	0.55	−0.19*	−0.19*	0.58***									
	Arabs	3.91	0.39	−0.05	−0.04	0.32***									
Openness															
T1	Jewish	4.36	0.52	0.08	−0.03	−0.63***	−0.39***								
	Arabs	4.22	0.46	0.05	−0.00	−0.67***	−0.33***								
T2	Jewish	4.35	0.56	0.09	0.03	−0.43***	−0.63***	0.53***							
	Arabs	4.29	0.46	0.14*	−0.02	−0.22***	−0.54***	0.33***							
Universalism															
T1	Jewish	3.94	0.69	0.07	0.10	−0.10	−0.03	−0.32***	−0.20*						
	Arabs	4.11	0.50	0.05	−0.02	−0.01	0.13	−0.32***	−0.03						
T2	Jewish	3.94	0.61	0.04	0.17*	−0.15	−0.17*	−0.05	−0.27***	0.40***					
	Arabs	4.09	0.54	0.02	−0.05	0.08	0.24***	−0.134*	−0.43***	0.27***					
Power															
T1	Jewish	2.98	1.06	−0.21***	−0.09	−0.34***	−0.09	0.10	−0.02	−0.34***	−0.20*				
	Arabs	2.57	1.16	−0.20***	0.04	−0.34***	−0.16*	0.05	0.02	−0.36***	−0.21**				
T2	Jewish	2.94	0.97	−0.04	0.06	−0.07	−0.32***	−10	−0.02	−0.04	−0.24**	0.50***			
	Arabs	2.72	1.09	−0.29***	0.15*	−0.27***	−0.38***	0.12	−0.03	−0.31***	−0.33***	0.44***			
National ID															
T1	Jewish	4.83	1.32	−0.07	0.06	0.27***	0.23**	−0.13*	−0.06	−0.13*	−0.20*	−0.13*	−0.05		
	Arabs	3.12	1.66	−0.08	0.11	0.00	−0.12	−0.04	−0.02	−0.08	−0.12	0.13*	0.13		
T2	Jewish	4.91	1.11	−0.04	0.04	0.26**	0.25***	−0.13	−0.07	−0.04	−0.22**	−0.10	−0.14	0.46***	
	Arabs	3.52	1.46	−0.16*	−0.06	0.01	−0.05	−0.18**	0.00	0.08	−0.08	0.21**	0.13*	0.26***	

*National ID* National Identification, *T1* Time 1, *T2* Time 2

^*^p < 0.05; ^**^p < 0.01; ^***^p < 0.001

#### Associations between values and national identification across time among Jewish and Arab adolescents

Missing data ranged from 0.0% to 44.9%. Little’s MCAR test was significant, *χ*^*2*^ (213, *n* = 568) = 378.15, *p* < 0.001, indicating data might not be MCAR. Therefore, our estimation method was MLR.

A multiple-group model was conducted as a function of group (Jewish and Arab), and for each value, the constrained model was compared to the free-to-vary model. The models controlled for participants’ gender and reported SES (parents’ education). A chi-test comparison showed no significant differences for the openness-to-change (*Δχ*^2^(10) = 13.76, *p* = 0.18) and universalism values (*Δχ*^2^(11) = 10.62, *p* = 0.47) models, indicating there were no significant group differences for these values. Therefore, these models remained constrained. Significant group differences were found between the constrained model and the free-to-vary model for conservation (Δχ^2^(10) = 35.39, *p* < 0.001) and power (*Δχ*^2^(6) = 19.29, *p* = 0.004) values. After the cross-lagged and concurrent paths were released in the models for these values, no differences were found between the partially constrained models and the free-to-vary models for conservation values (*Δχ*^*2*^(5) = 2.6, *p* = 0.79) or power values (*Δχ*^*2*^*(*6) = 2.52, *p* = 0.87). The final models reached good to excellent fit (see Kline, 2011): CFI = 0.96, RMSEA = 0.04, SRMR = 0.04 for conservation values; CFI = 0.90, RMSEA = 0.04, SRMR = 0.08 for openness-to-change values; CFI = 0.99, RMSEA = 0.01, SRMR = 0.067 for universalism values; CFI = 0.96, RMSEA = 0.04, SRMR = 0.05 for power values.

Table 4 presents the results for the path models of the cross-lagged associations between values and national identification for Jewish and Arab adolescents. For Jewish adolescents, in line with the first hypothesis, conservation values were associated with future national identification, as was similarly found in Study 1. Thus, adolescents who valued stability, conformity and tradition were more likely to increase their identification with the national group. A concurrent positive association between conservation values and national identification was also found at T1. In line with the second hypothesis, openness-to-change values were related negatively to future identification. That is, majority adolescents who valued new experiences and independent thought and action were less likely to show national group identification over time. In line with the third hypothesis, a cross-lagged negative association was found between universalism and national identification, but from identification to values. That is, Jewish adolescents, who tended towards national identification also tended to care less, over time, for social justice and for those in the outgroup. In addition, concurrent negative associations between national identification and universalism values appeared at T1 and T2. Finally, power values were concurrently negatively associated with national identification at T1 and T2.

**Table 4:** Models results linking values to national identification across times 1 and 2 among Jewish and Arab adolescents: Study 2

	Conservation				Openness				Universalism				Power			
	Jewish		Arabs		Jewish		Arabs		Jewish		Arabs		Jewish		Arabs	
	β	SE	β	SE	β	SE	β	SE	β	SE	β	SE	β	SE	β	SE
Auto-regressive paths																
Values T1-> Values T2	0.57***	0.07	0.31***	0.08	0.42***	0.05	0.41***	0.04	0.40***	0.06	0.31***	0.04	0.49***	0.04	0.46***	0.04
National Identification T1-> National identification T2	0.34***	0.06	0.31***	0.05	0.34***	0.06	0.31***	0.05	0.37***	0.05	0.33***	0.05	0.34***	0.06	0.30***	0.05
Cross-lagged relations																
Values T1 -> National Identification T2	0.20*	0.09	0.02	0.07	−0.13*	0.06	−0.08*	0.04	0.01	0.07	0.006	0.04	−0.09	0.08	0.14*	0.07
National identification T1 -> Values T2	0.08	0.07	−0.09	0.07	−0.02	0.04	−0.03	0.05	−0.1*	0.04	−0.13*	0.05	0.01	0.07	0.07	0.06
Concurrent relations																
Values T1 <-> National Identification T1	0.27***	0.05	0.01	0.06	−0.08	0.04	−0.07	0.04	−0.10*	0.04	−0.1*	0.04	−0.12**	0.04	0.08*	0.04
Values T2 <−> National Identification T2	0.03	0.06	0.03	0.05	0.03	0.06	0.03	0.05	−0.14*	0.06	−0.1*	0.04	−0.19**	0.06	0.10*	0.05

*Openness* openness-to-change values, *T1* Time 1, *T2* Time 2

^*^p < 0.05; ^**^p < 0.01; ^***^p < 0.001

Among the minority Arab adolescents, openness-to-change values were negatively associated with future national identification (in line with the second hypothesis), and power values were positively associated (in line with the fourth hypothesis) with future national identification. Thus, Arab adolescents who did not seek change and autonomy but aspired for status and control of people and resources were more likely to increase their identification with the national group over time. Positive associations for power values were found concurrently at T1 and T2. In line with the third hypothesis, cross-lagged negative associations were found for universalism values, between national identification and future universalism. Negative associations for universalism values appeared concurrently at T1 and T2 The analysis was rerun without the controls, with similar findings.

## Discussion

Establishing a collective sense of identification is vital for adolescents, as it plays a pivotal role in their well-being and nurtures a feeling of connection. Nevertheless, there is a lack of attention towards understanding the factors that impact national identification and the variations across diverse youth contexts. Two longitudinal studies examined the associations between values and national identification among two groups of adolescents: majority (Jewish Israelis) and minority (Arab citizens of Israel). The hypotheses were generally supported. In line with the first hypothesis, conservation values related to future national identification for the majority group in both studies. The second hypothesis was partially supported, in that openness-to-change values were negatively related to future national identification in the majority group but only in Study 2. The same relations appeared in Study 2 for the minority group. The third hypothesis on negative associations between universalism values and national identification was supported in both studies, with differences in directivity: there were significant negative associations between values and future national identification in Study 1; the same association appeared in Study 2, but the directivity was reversed, from national identification to future values. Finally, the fourth hypothesis was confirmed; power values were related to future national identification in the minority group (Study 2). As a result, our study has empirical evidence about the connection of two dimensions of identities: personal (i.e., values) and collective (i.e., national identification) (Eccles, 2009; Schwartz et al., 2008) in both adolescent samples. Moreover, values played a role in motivating national identification over time.

Although values seem to be a significant force in adolescents’ national identification, national identification may derive from different motivations in different groups, because context-specific effects were found for conservation and power values. There were, however, also motivationally similar effects for others values, openness-to-change and universalism values showed similar associations to national identification across groups. These findings may set the stage to assess group- or culture-specific motivations to feel part of the majority group, along with common or universal motivations.

With respect to the context-specific motivations, among the majority Jewish adolescents, national identification was associated with the current status quo. Conservation values represent a focus on the conservation of existing conditions and structures and adherence to ingroup expectations and norms. Thus, national identification may serve as means to fulfill the need of majority adolescents to feel safe, honor the past, maintain continuity, and belong to a large collective. It is worth noting that results for majority youth were very similar in the two studies – even though the studies used independent samples, slightly different measures, and different time intervals. This built-in longitudinal replication underscores the robustness of the findings for majority adolescents.

In contrast to their majority comparators, minority Arab adolescents’ conservation values were not associated with their national identification. Those adolescents may not see national identification as providing safety; nor does it signify a tradition they identify with or offer them a sense of belonging. The ongoing Israeli-Palestinian conflict and discrimination and rejection by the majority group (Hammack, 2010) may challenge the feeling of safety and stability that group identification might enhance.

The results further suggested that the motivation for national identification relates to additional values dimensions among minority adolescents - the contrast between self-enhancement and self-transcendence. Specifically, as hypothesized, power values were positively associated with national identification over time among minority Arab adolescents. One approach to enhancing one’s social identity status involves pursuing individual mobility, which might entail trying to assimilate into the dominant group (Tajfel & Turner, 1986). This strategy was found in a previous qualitative study among Arab adolescent citizens of Israel (Hammack, 2010). In a dominant majority culture, minority adolescents may increase their national identification as they struggle to realize their ambitions for status, impact, and control over resources. These findings support the idea that identification as Israeli is a prerequisite for Arabs to succeed in Israeli society. This might be a reflection of the Israeli assimilative atmosphere, which pushes minorities to blend into the larger society (Sher-Censor et al., 2018),although studies in other contexts have shown that an integration approach (i.e., combining the minority and majority cultures) often is more beneficial for minority adolescents’ psychosocial adaptation (Berry et al., 2006).

In addition to finding culture-specific motivations for national identification, the research also found shared motivations. In Study 2, openness-to-change values were negatively related to future national identification among both majority Jewish and minority Arab adolescents. Adolescents who attribute importance to new experiences, and freedom of thought and behavior may find identification with a big social group such as a nation hinders their independence and individuality. This process might be more prominent during adolescence in general, when youth strive for self-reliance and independence from adult figures to build a unique identification (Koepke & Denissen, 2012). It is important to note that these associations were not found for the majority group in Study 1, indicating the need for future studies to examine these relations.

The hypothesis about longitudinal negative association between universalism values and national identification was supported for both majority Jewish and minority Arab adolescents. It is important to note, however, that although negative associations were found in both studies, the longitudinal direction of effects between universalism values and national identification differed between studies. In Study 1, there was a cross-lagged negative association from universalism values at T1 to national identification at T2, but in Study 2, national identification at T1 predicted a decrease in universalism values at T2.

The cross-lagged design of the studies allowed the examination of the mutual relations between these factors. It might be that a mutual process occurred in two directions. Adolescents who attributed importance to tolerance, equality, and social justice seemed less likely to increase their national identification. Nevertheless, there was also evidence that adolescents who expressed national identification developed patriotic values emphasizing ingroup benefits. Over time, they seemed likely to care less for outgroups, including minority groups, a result in line with findings that right-wing voters endorse power values and not universalism values (Sagiv & Schwartz, 2022). The directivity difference between the studies may also reflect the age of the participants. Participants in Study 1 were older (approximately 16 years at T1) than Study 2 participants (approximately 13 years at T1). Older adolescents may already have achieved much of their personal identity formation goals, formed social commitments, and be less affected by other social partners, such as peer groups. Therefore, their group identification has a weaker effect. Younger adolescents are in the midst of this process. Given these observations, a promising research direction would be to combine the values-group perspective with a perspective on developmental needs at specific ages. The present results require more research and have to be replicated in more studies, across more time points, and with varying time intervals.

Importantly, the cross-lagged results pointed to more consistent directions from values to later national identification, than from national identification to later values. In other words, it seems that once adolescents define what is important to their personal identity, they may look for groups that enable the manifestation of this identification and thus develop their group identification. Values are abstract constructs, rather stable over time, and they function as an integral part of identity (Sagiv et al., 2017). Past studies found values were more likely to predict social behavior than vice versa (Benish-Weisman, 2015; Vecchione et al., 2016). Similar to behavior, national identification (as a surface-level cultural facet) is a less stable structure than values (deep cultural facets), and for that reason, may be easier to change (Hall, 1976).

The present results raise the question of which values society wants to encourage in young people. Many societies see national identification as a coveted quality, providing unity, a sense of meaning in life, and guidance (Idris et al., 2012; Johnston et al., 2010) and therefore apply educational efforts to strengthen it. To this, the studies add the caveat that values education aimed at achieving this goal may have side effects. In particular, conservation values among majority youth were previously associated with factors such as increased intolerance (Ben-Nun Bloom & Bagno-Moldavsky, 2015). The findings suggest that national identification relates to less emphasis on human rights and social justice. Moreover, strengthening the national identification of minority adolescents may alienate these adolescents from their ethnic communities and their families, resulting in long-term adjustment challenges (Nair et al., 2018). In addition, if different values are associated with national identification in majority and minority youth, an increase in national identification in general may divide societies’ values structure, with iatrogenic effects on national unification. More research is needed on outcomes of values and national identification to gain a complete picture of the role of values in socialization and what side effects are to be expected.

The research has a number of notable strengths. The longitudinal design is a gold standard in developmental research (Baltes et al., 2014). By following the same adolescents over time, the research provides information on developmental patterns and enables a focus on the direction of effects. Most results in the majority Jewish group were replicated across two samples, using different research methods, somewhat different age groups, and different time gaps. Thus, the results are likely to be robust. Moreover, the cross-cultural, comparative nature of Study 2 permitted the investigation of group-specific effects on the association between values and national identification.

At the same time, the work has some limitations. First, studies with only two time points limit the ability to assume causality. Longitudinal studies with at least three time points can provide more robust evidence by allowing the assessment of temporal relationships between variables over time (Gershoff et al., 2009). Moreover, cross-lagged path analysis was recently criticized (Hamaker et al., 2015), and extensions, such as random intercept models (Mulder & Hamaker, 2021), are increasingly popular. Unfortunately, the two-wave design did not permit this procedure, but the two-study design with replications and the moderate stability in the constructs suggest reliable findings. Nevertheless, future research may profit from more points of measurement allowing the differentiation of within-person processes from stable between-person differences through the inclusion of random intercepts (Hamaker et al., 2015).

Second, the effect sizes (as shown by the betas) are rather small. Such effects are to be expected in a field study among adolescents who face numerous simultaneous socialization demands from parents, peers, and the larger society (Titzmann et al., 2023). Moreover, research on effect sizes has shown that even small effects have practical relevance (McCartney & Rosenthal, 2000). For example, voting behavior may be affected, and in some instances, a few votes might suffice to cause major changes.

Third, there were relatively high levels of attrition in both studies, especially in Study 1, and there were several significant differences between the participants who completed both assessments and those who dropped out after the first assessment. Therefore, the results should be interpreted based on these findings. For example, in Study 2, participants who expressed a national identification with Israel and attributed importance to power values were more likely to drop out. Thus, there were fewer findings for them in the second assessment, thus possibly biasing or hindering the association between power values and national identification, especially among Jewish majority participants (who tended to have a stronger national identification with Israel, M_Jewis_h = 4.8 M_Arab_ = 3.5 *t(529)* = 13.79, *p* < 0.001).

Fourth, Israel is a very specific environment, featuring ongoing conflict. In less conflictual contexts, there may be no differences between majority and minority youth, or other values may be associated with national identification. One way to further our knowledge would be to conduct comparative studies in other contexts, compare other minority groups within the same context, or undertake qualitative research to gain deeper insight into what minority and majority adolescents associate with the national and/or cultural context. Such studies would provide a more nuanced view of the conditions, times, domains, and processes (Bornstein, 2017) that influence the association between values and national identification.

## Conclusion

Developing a sense of collective identification is crucial for adolescents, as it contributes to their well-being and fosters a sense of connection. However, there has been a lack of focus on understanding the factors influencing national identification and how they vary in different youth contexts.This research demonstrated the longitudinal associations between values and national identification among adolescents. While this finding held for both majority and minority youth, the association differed across groups. Majority adolescents tending towards national identification attributed more importance to conservation values, possibly to enhance their sense of stability and safety by being part of a group, while minority adolescents endorsed power values, possibly because they wanted to climb the social ladder and improve their social status. For both groups, national identification was related to less openness to new experiences and less care to people outside their ingroup. These are key findings, given today’s increasingly multicultural societies. Policymakers should take the findings into consideration when planning policies to enhance national identification. Programs aiming to enhance a cohesive society may need to be tailored to specific populations, because a one-size-fits-all developmental stance seems increasingly less conducive to creating a society where everybody can prosper and thrive.

## References

[CR1] Baltes, P. B., Reese, H. W., & Nesselroade, J. R. (2014). *Life-span developmental psychology: Introduction to research methods*. Psychology Press.

[CR2] Bar-Tal, D. (1993). Patriotism as fundamental beliefs of group members. *Politics and the Individual*, *3*(2), 45–62.

[CR3] Ben-Lulu, E., & Feldman, J. (2022). Reforming the Israeli–Arab conflict? Interreligious hospitality in Jaffa and its discontents. *Social Compass*, *69*(1), 3–21.

[CR76] Berry, J. W., Phinney, J. S., Sam, D. L. & Vedder, P. (2006). Immigrant youth: Acculturation, identity, andadaptation. *Applied Psychology*, *55*(3), 303–332.

[CR4] Ben-Nun Bloom, P., & Bagno-Moldavsky, O. (2015). The conditional effect of network diversity and values on tolerance. *Political Behavior*, *37*(3), 623–651. 10.1007/s11109-014-9284-2.

[CR5] Benish-Weisman, M. (2015). The interplay between values and aggression in adolescence: A longitudinal study. *Developmental Psychology*, *51*(5), 677–687. 10.1037/dev0000015. 25844848 10.1037/dev0000015

[CR6] Benish-Weisman, M. (2016). Brief report: Ethnic identity and aggression in adolescence: A longitudinal perspective. *Journal of Adolescence*, *47*, 131–134. 10.1016/j.adolescence.2015.05.015. 26113492 10.1016/j.adolescence.2015.05.015

[CR7] Bentler, P. M. (1990). Comparative fit indexes in structural models. *Psychological Bulletin*, *107*, 238–246. 10.1037/0033-2909.107.2.238. 2320703 10.1037/0033-2909.107.2.238

[CR8] Bornstein, M. H. (2017). The specificity principle in acculturation science. *Perspectives on Psychological Science*, *12*(1), 3–45. 10.1177/1745691616655997. 28073331 10.1177/1745691616655997 PMC5234695

[CR9] Branje, S. (2018). Development of parent–adolescent relationships: Conflict interactions as a mechanism of change. *Child Development Perspectives*, *12*(3), 171–176.

[CR10] Bubeck, M., & Bilsky, W. (2004). Value structure at an early age. *Swiss Journal of Psychology*, *63*(1), 31–41. 10.1024/1421-0185.63.1.31.

[CR11] Correll, J., & Park, B. (2005). A model of the ingroup as a social resource. *Personality and Social Psychology Review*, *9*(4), 341–359. 10.1207/s15327957pspr0904_4. 16223356 10.1207/s15327957pspr0904_4

[CR12] Crone, E. A., & Van Der Molen, M. W. (2007). Development of decision making in school‐aged children and adolescents: Evidence from heart rate and skin conductance analysis. *Child Development*, *78*(4), 1288–1301. 10.1111/j.1467-8624.2007.01066.x. 17650139 10.1111/j.1467-8624.2007.01066.x

[CR13] Daniel, E., Benish-Weisman, M., Boehnke, K., & Knafo, A. (2016). Personal and culture-dependent values as part of minority adolescent identity. In *The Challenges of Diaspora Migration* (pp. 103–126). Routledge.

[CR14] Dumontheil, I., & Blakemore, S. J. (2012). Social cognition and abstract thought in adolescence: The role of structural and functional development in rostral prefrontal cortex. *British Journal of Educational Psychology Monograph Series II*, *8*, 99–113.

[CR15] Eccles, J. (2009). Who am I and what am I going to do with my life? Personal and collective identities as motivators of action. *Educational Psychologist*, *44*(2), 78–89. 10.1080/00461520902832368.

[CR16] Feasel, S. H., Risen, J. L., & White, S. M. (2021). Tied to both sides or asserting a preferred identity? The case of Palestinian citizens of Israel in an intergroup contact setting. *Self and Identity*, *20*(4), 545–568. 10.1080/15298868.2019.1634143.

[CR17] Fine, M., & Sirin, S. R. (2007). Theorizing hyphenated selves: Researching youth development in and across contentious political contexts. *Social and Personality Psychology Compass*, *1*(1), 16–38. 10.1111/j.1751-9004.2007.00032.x.

[CR18] Gershoff, E. T., Aber, J. L. & Clements, M. (2009). Parent learning support and child reading ability: A cross-lagged panel analysis for developmental transactions. In A. J. Sameroff (Ed.), *The transactional model of development. How children and contexts shape each other* (pp. 203–220). American Psychological Association. 10.1037/11877-011.

[CR19] Greenberg, J., Pyszczynski, T., Solomon, S., Rosenblatt, A., Veeder, M., Kirkland, S., & Lyon, D. (1990). Evidence for terror management theory II: The effects of mortality salience on reactions to those who threaten or bolster the cultural worldview. *Journal of Personality and Social Psychology*, *58*(2), 308–318. 10.1037/0022-3514.58.2.308.

[CR75] Hall, E. T. (1976). Beyond culture. Anchor.

[CR20] Hamaker, E. L., Kuiper, R. M., & Grasman, R. P. P. P. (2015). A critique of the cross-lagged panel model. *Psychological Methods*, *20*(1), 102–116. 10.1037/a0038889. 25822208 10.1037/a0038889

[CR21] Hammack, P. L. (2010). Narrating hyphenated selves: Intergroup contact and configurations of identity among young Palestinian citizens of Israel. *International Journal of Intercultural Relations*, *34*(4), 368–385. 10.1016/j.ijintrel.2010.03.002.

[CR22] Havighurst, R. J. (1972). *Developmental task and education*. David McKay Company Inc.

[CR23] Hogg, M. A., & Abrams, D. (2007). Intergroup behavior and social identity. In M. A. Hogg & J. Cooper (Eds.), *The Sage handbook of social psychology: Concise student edition* (pp. 335–360). Sage.

[CR24] Horenczyk, G., & Ben-Shalom, U. (2006). Acculturation in Israel. In D. L. Sam & J. W. Berry (Eds.), *The Cambridge handbook of acculturation psychology* (pp. 294–310). Cambridge University Press. 10.1017/CBO9780511489891.023.

[CR25] Hu, L. T., & Bentler, P. M. (1999). Cutoff criteria for fit indexes in covariance structure analysis: Conventional criteria versus new alternatives. *Structural Equation Modeling*, *6*, 1–55. 10.1080/10705519909540118.

[CR26] Idris, F., Hassan, Z., Ya’acob, A., Gill, S. K., & Awal, N. A. M. (2012). The role of education in shaping youth’s national identity. *Procedia-Social and Behavioral Sciences*, *59*, 443–450. 10.1016/j.sbspro.2012.09.299.

[CR27] Israel Central Bureau of Statistics. (2022). *Statistical abstract of Israel 2021*. https://www.cbs.gov.il/en/Pages/search/yearly.aspx.

[CR28] Johnston, R., Banting, K., Kymlicka, W., & Soroka, S. (2010). National identity and support for the welfare state. *Canadian Journal of Political Science/Revue canadienne de science politique*, *43*(2), 349–377. 10.1017/S0008423910000089.

[CR29] Jugert, P., Leszczensky, L., & Pink, S. (2018). The effects of ethnic minority adolescents’ ethnic self‐identification on friendship selection. *Journal of Research on Adolescence*, *28*(2), 379–395. 10.1111/jora.12337. 28815988 10.1111/jora.12337

[CR30] Kenny, D. A. (1975). Cross-lagged panel correlation: A test for spuriousness. *Psychological Bulletin*, *82*, 887–903. 10.1037/0033-2909.82.6.887.

[CR31] Kiang, L., Yip, T., Gonzales‐Backen, M., Witkow, M., & Fuligni, A. J. (2006). Ethnic identity and the daily psychological well‐being of adolescents from Mexican and Chinese backgrounds. *Child Development*, *77*(5), 1338–1350. 10.1111/j.1467-8624.2006.00938.x. 16999802 10.1111/j.1467-8624.2006.00938.x

[CR32] Kline, R. B. (2011). *Principles and practice of structural equation modeling*. Guilford Press.

[CR33] Knafo, A., Daniel, E., & Khoury‐Kassabri, M. (2008). Values as protective factors against violent behavior in Jewish and Arab high schools in Israel. *Child Development*, *79*(3), 652–667. 10.1111/j.1467-8624.2008.01149.x. 18489419 10.1111/j.1467-8624.2008.01149.x

[CR34] Knafo, A., & Schwartz, S. H. (2003). Parenting and adolescents’ accuracy in perceiving parental values. *Child Development*, *74*(2), 595–611. 10.1111/1467-8624.7402018. 12705575 10.1111/1467-8624.7402018

[CR35] Koepke, S., & Denissen, J. J. (2012). Dynamics of identity development and separation–individuation in parent–child relationships during adolescence and emerging adulthood–A conceptual integration. *Developmental Review*, *32*(1), 67–88. 10.1016/j.dr.2012.01.001.

[CR36] Kroger, J. (2006). *Identity development: Adolescence through adulthood*. Sage.

[CR37] Kuhn, D. (2009). The importance of learning about knowing: Creating a foundation for development of intellectual values. *Child Development Perspectives*, *3*(2), 112–117. 10.1111/j.1750-8606.2009.00089.x.

[CR38] Kuhn, D., Cheney, R., & Weinstock, M. (2000). The development of epistemological understanding. *Cognitive Development*, *15*(3), 309–328. 10.1016/S0885-2014(00)00030-7.

[CR39] LaFromboise, T., Coleman, H. L., & Gerton, J. (1993). Psychological impact of biculturalism: evidence and theory. *Psychological Bulletin*, *114*(3), 395–412. 10.1037/0033-2909.114.3.395. 8272463 10.1037/0033-2909.114.3.395

[CR40] Laursen, B., & Veenstra, R. (2021). Toward understanding the functions of peer influence: A summary and synthesis of recent empirical research. *Journal of Research on Adolescence*, *31*(4), 889–907. 34820944 10.1111/jora.12606 PMC8630732

[CR41] Litvak-Hirsch, T., Bar-On, D., & Chaitin, J. (2003). Whose house is this? Dilemmas of identity construction in the Israeli-Palestinian context. *Peace and Conflict: Journal of Peace Psychology*, *9*(2), 127–148. 10.1037/dev0000318.

[CR42] Marsh, H. W., Balla, J. R., & McDonald, R. P. (1988). Goodness-of-fit indexes in confirmatory factor analysis: The effect of sample size. *Psychological Bulletin*, *103*, 391–410. 10.1037/0033-2909.103.3.391.

[CR43] McCartney, K., & Rosenthal, R. (2000). Effect size, practical importance, and social policy for children. *Child Development*, *71*(1), 173–180. 10.1111/1467-8624.00131. 10836571 10.1111/1467-8624.00131

[CR44] Mulder, J. D., & Hamaker, E. L. (2021). Three extensions of the random intercept cross-lagged panel model. *Structural Equation Modeling: A Multidisciplinary Journal*, *28*(4), 638–648. 10.1080/10705511.2020.1784738.

[CR45] Muthén, L. K., & Muthén, B. O. (2017). *Mplus user’s guide* (8th ed.). https://www.statmodel.com/html_ug.shtml.

[CR46] Nair, R. L., Roche, K. M., & White, R. (2018). Acculturation gap distress among Latino youth: Prospective links to family processes and youth depressive symptoms, alcohol use, and academic performance. *Journal of Youth and Adolescence*, *47*(1), 105–120. 10.1007/s10964-017-0753-x. 29030790 10.1007/s10964-017-0753-x PMC10352643

[CR47] Nguyen, A. M. D., & Benet-Martínez, V. (2013). Biculturalism and adjustment: A meta-analysis. *Journal of Cross-Cultural Psychology*, *44*(1), 122–159.

[CR48] Piurko, Y., Schwartz, S. H., & Davidov, E. (2011). Basic personal values and the meaning of left‐right political orientations in 20 countries. *Political Psychology*, *32*(4), 537–561. 10.1111/j.1467-9221.2011.00828.x.

[CR49] Roccas, S. (2003). Identification and status revisited: The moderating role of self-enhancement and self-transcendence values. *Personality and Social Psychology Bulletin*, *29*(6), 726–736. 10.1177/0146167203029006005. 15189628 10.1177/0146167203029006005

[CR50] Roccas, S., Sagiv, L., Schwartz, S., Halevy, N., & Eidelson, R. (2008). Toward a unifying model of identification with groups: Integrating theoretical perspectives. *Personality and Social Psychology Review*, *12*(3), 280–306. 10.1177/1088868308319225. 18641386 10.1177/1088868308319225

[CR51] Roccas, S., Schwartz, S. H., & Amit, A. (2010). Personal value priorities and national identification. *Political Psychology*, *31*(3), 393–419. 10.1111/j.1467-9221.2010.00763.x.

[CR52] Sagiv, L., Roccas, S., Cieciuch, J., & Schwartz, S. H. (2017). Personal values in human life. *Nature Human Behaviour*, *1*(9), 630–639. 10.1038/s41562-017-0185-3. 31024134 10.1038/s41562-017-0185-3

[CR53] Sagiv, L., & Schwartz, S. H. (2022). Personal values across cultures. *Annual Review of Psychology*, *73*, 517–546. 10.1146/annurev-psych-020821-125100. 34665670 10.1146/annurev-psych-020821-125100

[CR54] Schiefer, D., Möllering, A., Daniel, E., Benish‐Weisman, M., & Boehnke, K. (2010). Cultural values and outgroup negativity: A cross‐cultural analysis of early and late adolescents. *European Journal of Social Psychology*, *40*(4), 635–651. 10.1002/ejsp.745.

[CR55] Schwartz, S. H. (1992). Universals in the content and structure of values: Theoretical advances and empirical tests in 20 countries. In *Advances in experimental social psychology* (Vol. 25, pp. 1-65). Academic Press.

[CR56] Schwartz, S. H. (2005). Robustness and fruitfulness of a theory of universals in individual human values. In A. Tamayo & J. Porto (Eds.), *Valores e trabalho* [Values and work]. Editora Universidade de Brasilia.

[CR57] Schwartz, S. H. (2011). Studying values: Personal adventure, future directions. *Journal of Cross-Cultural Psychology*, *42*(2), 307–319. 10.1177/0022022110396925.

[CR58] Schwartz, S. H. (2012). An overview of the Schwartz theory of basic values. *Online Readings in Psychology and Culture*, *2*(1). 10.9707/2307-0919.1116.

[CR59] Schwartz, S. H., Caprara, G. V., & Vecchione, M. (2010). Basic personal values, core political values, and voting: A longitudinal analysis. *Political Psychology*, *31*(3), 421–452. 10.1111/j.1467-9221.2010.00764.x.

[CR60] Schwartz S. H., Lehman A., Roccas, S. (1999). Multimethod probes of basic human values. In J. Adamopoulos & Y. Kashira (Eds.), *Social psychology and culture context: Essays in honour of Harry C. Triandis* (pp. 107–123). Sage.

[CR61] Schwartz, S. H., Melech, G., Lehmann, A., Burgess, S., Harris, M., & Owens, V. (2001). Extending the cross-cultural validity of the theory of basic human values with a different method of measurement. *Journal of Cross-Cultural Psychology*, *32*(5), 519–542. 10.1177/0022022101032005001.

[CR62] Schwartz, S. J., Zamboanga, B. L., & Weisskirch, R. S. (2008). Broadening the study of the self: Integrating the study of personal identity and cultural identity. *Social and Personality Psychology Compass*, *2*(2), 635–651. 10.1111/j.1751-9004.2008.00077.x.

[CR63] Sellers, R. M., Rowley, S. A., Chavous, T. M., Shelton, J. N., & Smith, M. A. (1997). Multidimensional Inventory of Black Identity: A preliminary investigation of reliability and construct validity. *Journal of Personality and Social Psychology*, *73*(4), 805–815. 10.1037/0022-3514.73.4.805.

[CR64] Sher-Censor, E., Benish-Weisman, M., Gal, L., & Karni, S. (2018). The associations between national identity and adjustment: What can we learn from autobiographical narratives? *International Journal of Intercultural Relations*, *67*, 12–24. 10.1016/j.ijintrel.2018.08.003.

[CR65] Smith, T. B., & Silva, L. (2011). Ethnic identity and personal well-being of people of color: A meta-analysis. *Journal of counseling psychology*, *58*(1), 42–46. 10.1002/ejsp.745. 21171745 10.1037/a0021528

[CR66] Solomon, S., Greenberg, J., & Pyszczynski, T. (1991). A terror management theory of social behavior: The psychological functions of self-esteem and cultural worldviews. In *Advances in experimental social psychology* (Vol. 24, pp. 93–159). Academic Press. 10.1016/S0065-2601(08)60328-7.

[CR67] Tajfel, H. (1974). Social identity and intergroup behaviour. *Social Science Information*, *13*(2), 65–93. 10.1177/053901847401300204.

[CR68] Tajfel, H. & Turner, J. (1979). An integrative theory of intergroup conflict. In W. G. Austin & S. Worchel (Eds.), *The social psychology of intergroup relations*. Brooks/Cole.

[CR69] Tajfel, H., & Turner, J. C. (1986). The social identity theory of intergroup behavior. In S. Worchel & W. G. Austin (Eds.), *Psychology of intergroup relations* (pp. 7–24). Nelson-Hall.

[CR70] Telzer, E. H., Dai, J., Capella, J. J., Sobrino, M., & Garrett, S. L. (2022). Challenging stereotypes of teens: Reframing adolescence as window of opportunity. *American Psychologist*, *77*(9), 1067. 36595405 10.1037/amp0001109

[CR71] Titzmann, P. F., & Jugert, P. (Eds.). (2019). *Youth in superdiverse societies: Growing up with globalization, diversity, and acculturation*. Routledge.

[CR72] Titzmann, P. F., Paizan, M., A., & Aumann, L. (2023). Socialization. In *Elsevier reference collection in neuroscience and biobehavioral psychology*. Elsevier online. 10.1016/B978-0-323-96023-6.00038-5.

[CR73] Vecchione, M., Döring, A. K., Alessandri, G., Marsicano, G., & Bardi, A. (2016). Reciprocal relations across time between basic values and value‐expressive behaviors: A longitudinal study among children. *Social Development*, *25*(3), 528–547. 10.1111/sode.12152.

[CR74] Yip, T., Cheon, Y. M., & Wang, Y. (2019). The diversity paradox: Opportunities and challenges of ‘contact in context’ across development. *Research in Human Development*, *16*(1), 51–75. 10.1080/15427609.2018.1549404. 31588201 10.1080/15427609.2018.1549404 PMC6777864

